# Multicup reconstruction technique for the management of severe protrusio acetabular defects

**DOI:** 10.1186/s42836-021-00081-9

**Published:** 2021-07-06

**Authors:** Baochao Ji, Guoqing Li, Xiaogang Zhang, Yang Wang, Wenbo Mu, Li Cao

**Affiliations:** Department of Orthopaedics, the First Affiliated Hospital of Xinjiang Medical University, 137 South LiYuShan Road, 830054 Urumqi, Xinjiang China

**Keywords:** Revision, Total hip arthroplasty, Acetabular defect

## Abstract

**Background:**

In revision hip arthroplasty, managing the large protrusio acetabular defects remains a challenge. The report described a novel technique which employs a trabecular metal revision shell as a super-augment to buttress the superior medial structure.

**Methods:**

Between January 2015 and December 2018, the multicup reconstruction was performed in 21 patients with severe protrusio acetabular defects. The revision shell, plus two similar porous acetabular components was implanted into the initial shell to create a “multicup” construct. The functional outcomes were evaluated in terms of the Harris Hip Score. Acetabular loosening, restoration of hip center of rotation, and bone ingrowth etc., were radiographically assessed. The survival rate of the implants was also evaluated.

**Results:**

A followup lasting a mean time of 31 months (range, 18–57 months) revealed that the average Harris Hip Score improved from preoperative 37.0 ± 7.1 to postoperative 76.4 ± 9.0. There were no revisions due to acetabular loosening. The horizontal offset increased by an average of 14 mm, and the vertical offset decreased by an average of 18 mm. Eighteen of the 21 patients (86 %) met at least 3 of 5 criteria associated with bone ingrowth. The survivorship free from re-revision for acetabular loosening after 2 years was 100 %.

**Conclusions:**

The multicup reconstruction technique was a simplified re-revision procedure for managing the severe protrusio acetabular defects and could achieve a high survival rate.

**Level of evidence:**

Therapeutic study, Level IVa.

## Introduction

Patel et al. [1] estimated that, in England and Wales from 2012 to 2030, the number of total hip revisions will increase by 31 %. Revision hip arthroplasty poses a number of challenges, especially in patients with severe protrusio acetabular defects [2, 3].

The classification developed by Paprosky et al. [4] included severe protrusio acetabular defects (Paprosky IIC) and combined vertical bone loss (Paprosky IIIA, IIIB). The acetabular reconstruction is designed to restore the nearly normal hip mechanics and to provide the long-term stability of the acetabular component. The treatment includes the use of bulk allografts, antiprotrusio cages, cup-cages, custom triflanges, and trabecular metal (TM) cup plus augments. Bulk allografts taken from the femoral head are traditionally used to reconstruct the protrusio acetabular defects without pelvic discontinuity. Excellent results are achieved over a short period of time, but the failure rate was 47 % after 10 years [5]. The bone remodeling changes lead to graft resorption and finally contribute to acetabular loosening and failure of the reconstruction. The antiprotrusio cage is most widely used in difficult cases and attains good outcomes [6–9]. This technique is reliable in elderly patients. In younger patients who are physically more active, however, implant breakage is likely due to the lack of stability and biological fixation [10]. The cup-cage technique provides a reliable solution for the complex acetabular defects [11]. Using the customized triflange cups can achieve a long-lasting fixation with the acetabular component in the anatomic position [12]. The drawbacks include delays in surgery due to manufacturing, high cost, and the possibility of unexpected situations during surgery. Using the TM augment and shell can accomplish optimal results, but the augment may be cumbersome in the reconstruction of large protrusio acetabular defects involving the interior spatial structure [13]. Moreover, it is difficult or even impossible to orientate and fix the component through a narrow access. Using a TM-coated cup for augmentation often permits some degrees of interface fit and an enhanced surface area for bone ingrowth, but hip impingement is a major concern. For these reasons, we developed a new multicup reconstruction technique to simplify the surgery.

The purpose of the retrospective study was to review the a cohort of consecutive patients with major protrusio acetabular defects (Paprosky IIC, IIIB and IIIA) who had been treated with the multicup reconstruction. We used three kinds of cups (“cup-in-cup”, “cup-on-cup”, and “hybrid cup” techniques) on the basis of the size and location of the bone defects. This study answered 3 questions: (1) How to perform multicup reconstruction with the severe protrusio acetabular defects? (2) Will joint function improve in terms of the Harris Hip Score (HSS) after multicup reconstruction? and (3) What are the post-surgery radiographic outcomes?

## Patients and Methods

### Demographics of the patients

This study was approved by the institutional review board of our hospital. Using our institution-based registry database, we identified 274 patients who had undergone revision total hip arthroplasties (THAs) between January 2015 and December 2018. Among this cohort, 25 patients with severe protrusio acetabular defects received revision with the multicup reconstruction. Four patients were excluded because one died of unrelated disease and another 3 patients were lost to follow-up. Of the remaining 21 patients, 8 were female and 13 male. The mean age at the time of revision surgery was 56 years (range, 34–76 years) (Table 1). The causes for revision were aseptic loosening in 14 patients and chronic periprosthetic joint infection in 6 patients. Only 1 patient underwent reimplantation due to infection at the end of the first stage.

**Table 1:** Demographic factors for 21 episodes with severe protrusio acetabular defects treated by the multicup reconstitution technique

Patient No.	Gender	Age, y	Preoperative Diagnosis	Preoperative Comorbidities	Total or Partial Revision			Previous Operation
1	Male	42	Aseptic loosening	None	Total		THA	
2	Female	65	Reimplantation	Diabetes mellitus type II		Total	THA; Arthroplasty resection	
3	Male	41	Chronic PJI	None	Total		THA	
4	Male	76	Aseptic loosening	None	Total		THA; Revision for aseptic loosening	
5	Male	37	Aseptic loosening	None	Total		THA	
6	Female	55	Aseptic loosening	None	Total		THA	
7	Male	49	Aseptic loosening	None	Only acetabular revision		THA	
8	Male	76	Chronic PJI	Hypertension	Total		THA	
9	Male	60	Chronic PJI	Hypertension	Total		ORIF; THA; Debridement	
10	Male	34	Aseptic loosening	None	Total		THA	
11	Female	61	Chronic PJI	None	Total		THA	
12	Female	58	Aseptic loosening	None	Only acetabular revision		THA	
13	Female	49	Aseptic loosening	None	Only acetabular revision		THA	
14	Male	46	Chronic PJI	None	Total		THA; Debridement*2	
15	Female	68	Aseptic loosening	Rheumatoid arthritis	Only acetabular revision		THA; Revision for aseptic loosening	
16	Male	35	Aseptic loosening	None	Only acetabular revision		THA	
17	Female	69	Aseptic loosening	None	Total		THA	
18	Male	67	Aseptic loosening	None	Total		THA	
19	Female	74	Chronic PJI	Diabetes mellitus type II; Hypertension; Coronary artery disease	Total		THA; Debridement*3	
20	Male	53	Aseptic loosening	Hypertension	Total		THA	
21	Male	68	Aseptic loosening	None	Only acetabular revision		THA	

*PJI* periprosthetic joint infection; *THA* total hip arthroplasty; *ORIF* open reduction internal fixation

The acetabular deficiencies were radiographically classified preoperatively and confirmed intraoperatively by a senior surgeon against the Parprosky classification [4]. Preoperative pelvic discontinuity was excluded by using CT scan. According to the records, 9 were diagnosed as Paprosky IIC, and 12 as Paprosky IIIB bone deficiencies.

### Indications and Contraindications of Multicup Reconstruction

The indications of the multicup reconstruction were (1) severe protrusio acetabular defects (Paprosky IIC) or combined vertical bone loss (Paprosky IIIB); and (2) sufficient medial host bone available for achieving a stable press-fit fixation of the medial tantalum acetabular shell. The contraindications included (1) the presence of pelvic discontinuity after explantation; (2) It was impossible to achieve a stable press-fit fixation of the medial acetabular shell due to inadequate bone stock.

**Fig. 1:**
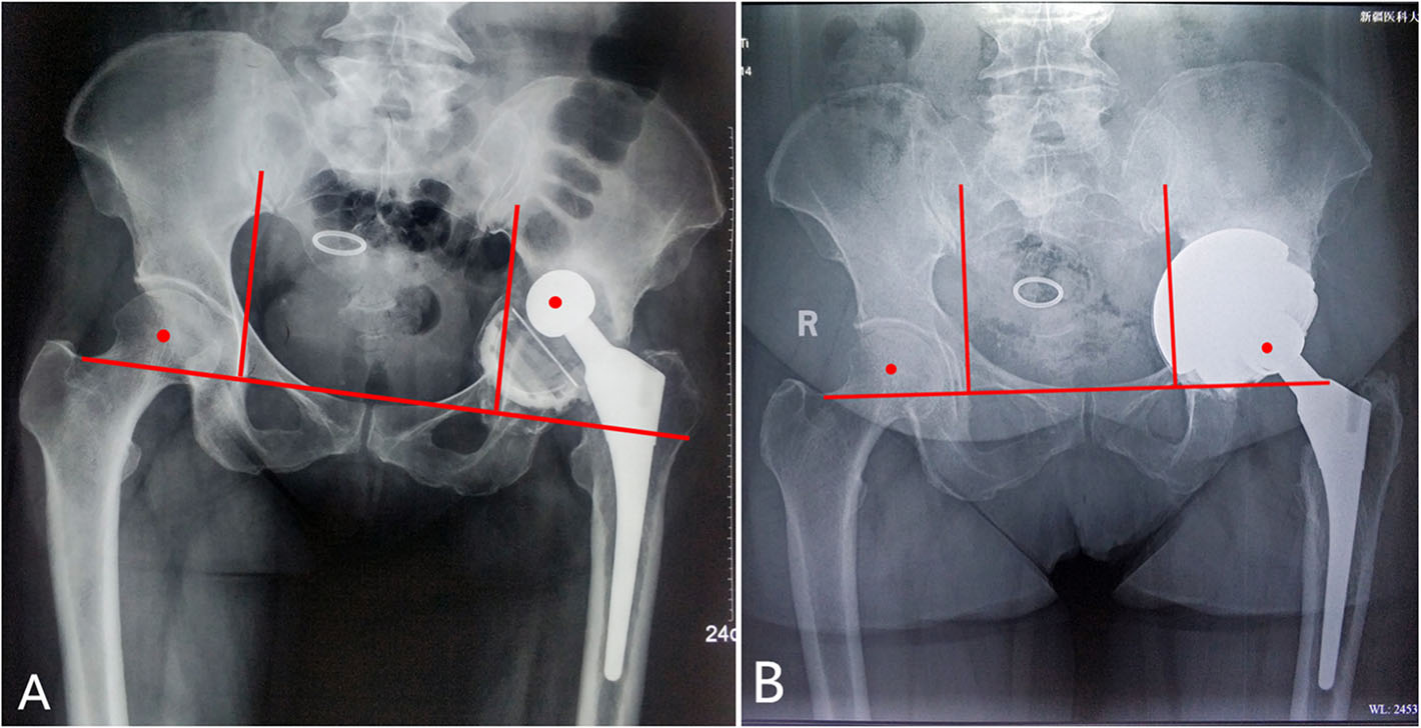
A 60-year-old female patient sustained a loosening cup. **A** A Paprosky IIC defect. **B** The “cup-in-cup” reconstruction technique with 3 porous shells (out diameters of the shells from medial to lateral: 70 mm; 64 mm; and 58 mm). The center of rotation of the hip was restored

**Fig. 2:**
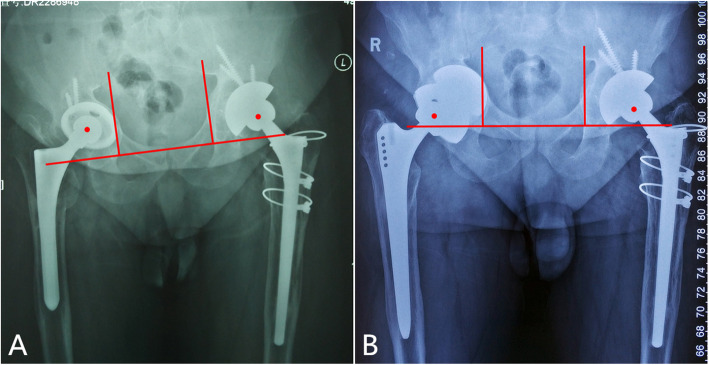
A 51-year-old male patient who had previous failure of a total hip arthroplasty. **A** A Paprosky IIC defect on the right hip. **B** The “cup-on-cup” reconstruction technique was performed with 2 tantalum TM shells. The larger tantalum cup was impacted onto the medial TM revision shell and a rigid fixation was achieved based on the lateral acetabular rim (out diameters of the shell from medial to lateral: 52 mm and 58 mm)

**Fig. 3:**
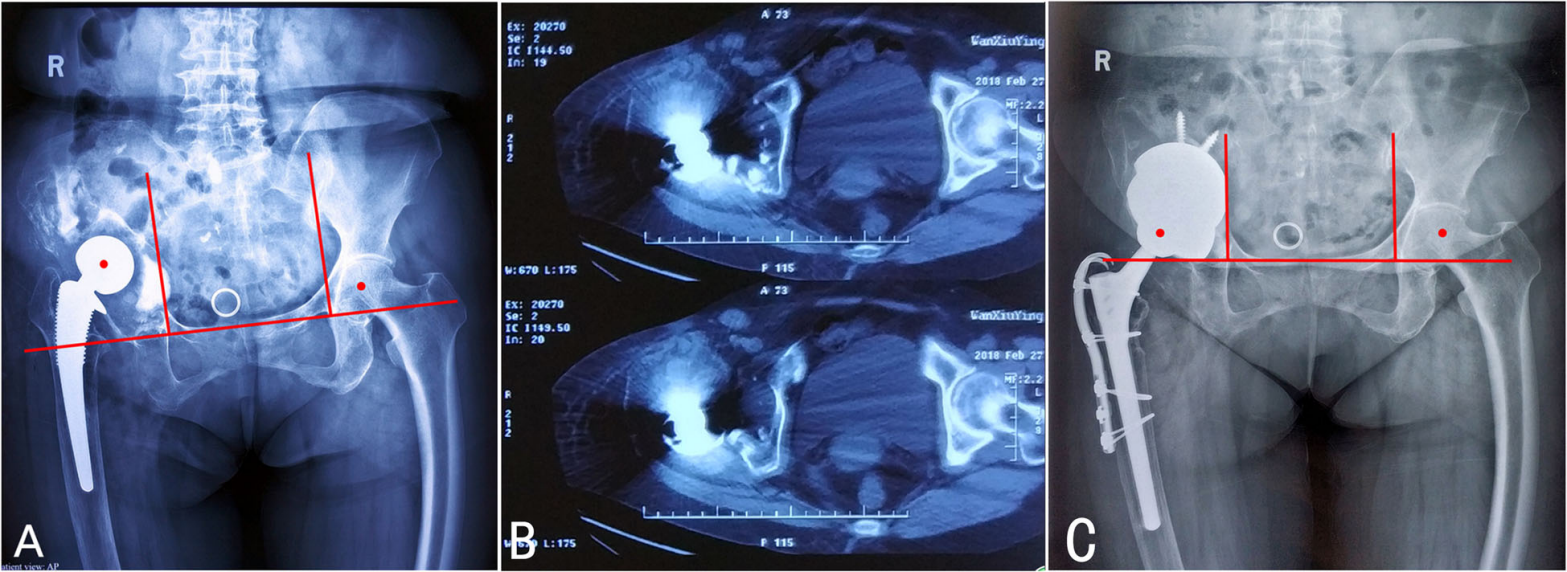
A 66-year-old female patient who had an infected total hip arthroplasty. **A** The preoperative X-ray showing a Paprosky IIIB defect. **B** The pelvic continuity was confirmed on CT scans. **C** The “hybrid cup” reconstruction technique was used with 3 porous shells. The smaller middle cup was eccentrically cemented into the medial TM revision shell to fill the sideward defects. A larger porous cup was then impacted onto the “cup-in-cup” construct (out diameters of the shell from medial to lateral: 66 mm; 54 mm; and 62 mm)

The surgical procedures included “cup-in-cup”, “cup-on-cup” and “hybrid cup” techniques according to the relationships between the cups. (1) The “cup-in-cup” technique was used for Parprosky IIC or IIIB defects with the loss of lateral support. After impacting a TM revision shell, a smaller porous cup was cemented into the shell combined with a modular liner to reconstruct the COR of the hip (Fig. 1 A, B). (2) The “cup-on-cup” technique was employed in Parprosky IIC or IIIB defects with adequate lateral support for three-point fixation. A larger porous cup was impacted onto the medial TM revision shell and was rigidly fixed by a lateral acetabular rim (Fig. 2 A, B). (3) The “hybrid cup” technique was performed for Parprosky IIIB defects with severe multiorientational bone defects that required a three-cup reconstruction. This surgery combined the “cup-in-cup” and “cup-on-cup” techniques. Even if the lateral acetabular rim was available for impaction, a smaller middle cup was still needed to pour eccentric cement into the medial TM revision shell, to fill the sideward defects. A larger porous cup was then impacted onto the “cup-in-cup” construct (Fig. 3 A-D).

### Surgical Technique

**Fig. 4:**
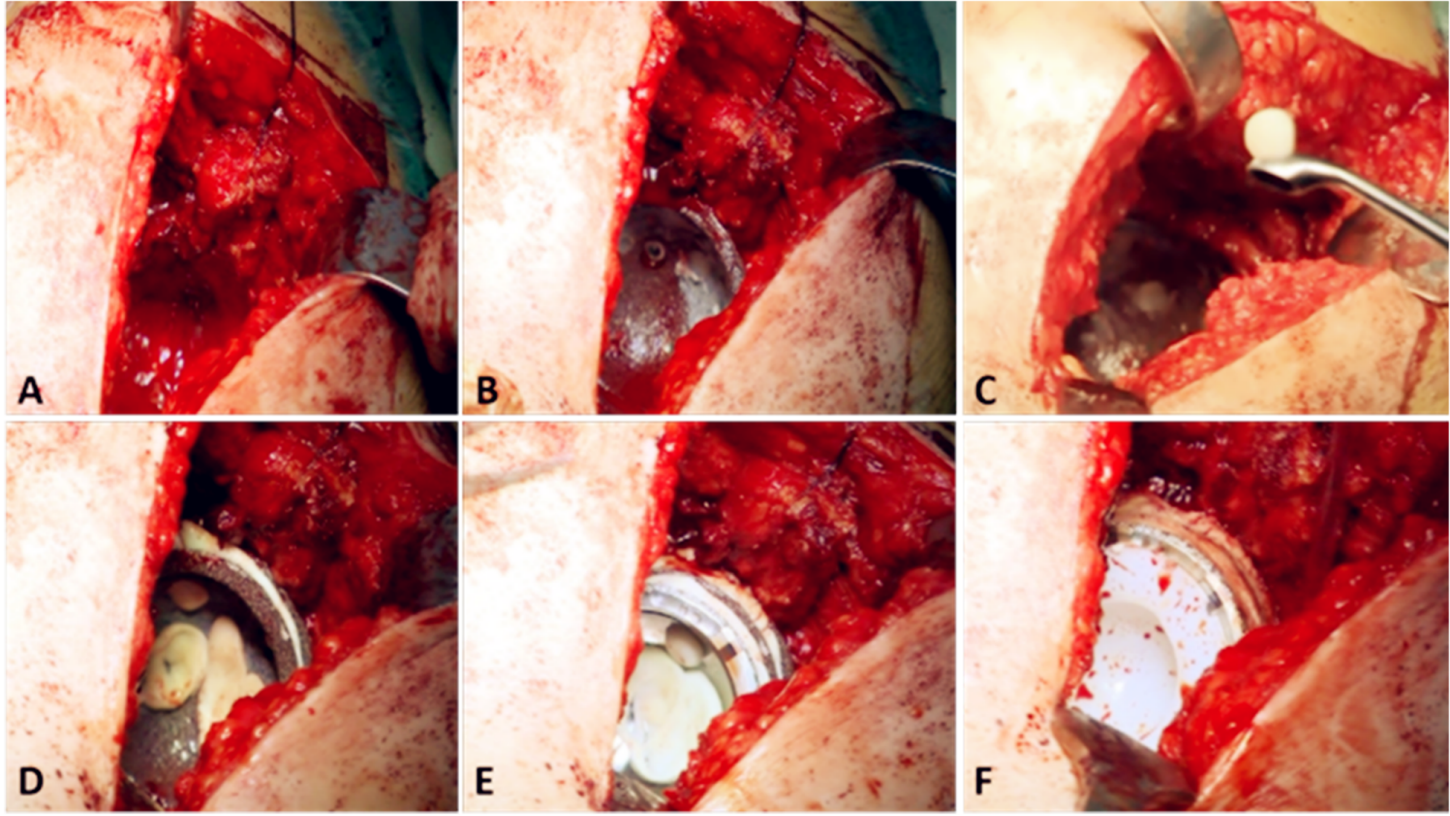
Intraoperative photographs demonstrating the “multicup” technique. **A** After removal of the failed acetabular components, cement and membrane, the acetabular deficiencies were confirmed again intraoperatively. **B** The medial TM tantalum revision shell was implanted into the medial defect with multiple screws to serve as upgraded-augment or the foundation for the following porous cups. **C** The screw holes on the bottom TM cup were blocked with wax to prevent the cement from entering the interface between the host bone and the tantalum revision shell. **D** The smaller second cup was cemented into the medial shell with the desired anteversion and abduction. **E** The additional porous cup was added to the finished “cup-in-cup” construct. **F** The modular liner with a 10° elevated rim was inserted

All operations were done by the same senior surgeon who is experienced in hip revision. The operation was performed through the postero-lateral approach. After removal of the failed acetabular components, cement and membrane, the acetabular defect was re-assessed intraoperatively (Fig. 4 A). The acetabulum was sequentially reamed to transform the cavity of the bone defect into a hemispherical shape and achieve the maximal engagement of the cup trial with the host pelvic bone. Line-to-line reaming was performed for Paprosky IIC and IIIA defects. For Paprosky IIIB defects, the bone bed was prepared for impaction with under-reaming (4 mm). The reaming debris was retained for autologous bone grafting during the later stage. Then, the medial host bone and the lateral acetabular ring were assessed for selecting the optimal reconstruction technique. Thereafter, the acetabular defect was sized, and cup trials were placed to bring the COR to the anatomic position. The acetabular anterior and posterior edges were used as the reference landmarker of horizontal offset. The acetabular inferior edge was chosen as the reference landmarker of vertical offset. The autografts were placed in the bottom of acetabulum, and the medial TM tantalum revision shell (Zimmer, Warsaw, IN) was implanted into the superior or medial defect to serve as a upgraded augment or the foundation for the following porous cups (Fig. 4B). In order to achieve a rigid fixation, the bottom cup was secured with several screws. In order to achieve a long-term biological fixation, the unused screw holes on the bottom of TM cup were filled with bone wax to prevent the cement extrusion through the holes to the interface between the host bone and the tantalum revision shell (Fig. 4 C). Once the medial TM tantalum cup was implanted, a smaller second cup (at least 4 mm smaller in diameter) was cemented into the shell with the desired anteversion and abduction based on the re-evaluated anatomic hip center as confirmed by using the remaining acetabular landmarks (Fig. 4D). The hip was reduced with an appropriately-sized liner trial.

The COR of hip joint was defined as the COR of the femur from the pelvis in all directions. If there was an impingement due to a failed attempt to restore the anatomical rotation center with the two cups, an additional porous cup (at least 4 mm smaller in diameter) was inserted to restore the offset (“cup-in-cup” construct) (Fig. 4E). If the lateral rim of acetabulum was intact with adequate bone stock remaining, a larger tantalum cup (Zimmer, Warsaw, IN) was impacted onto the “cup-in-cup” construct to achieve a reliable press-fit fixation between the host bone and implant. The lateral surface of the cup was secured with Trilogy cancellous screws (Zimmer, Warsaw, IN). The interspace between the middle and lateral cups was filled with cement. Otherwise, a smaller porous cup with a modular liner was cemented into the “cup-in-cup” construct to reconstruct the COR of the hip. A liner with a 10°-elevated rim was inserted to optimize the combined anteversion of the cup and stem in all cases (Fig. 4 F).

### Postoperative Rehabilitation

Within 6 weeks after surgery, the patients were allowed to walk with toe-touch weight bearing (limited to within the room). Partial weight bearing was allowed for another 6 weeks, and followed by full weight bearing. Most of the patients were allowed to walk without crutches and with full weight bearing after 4 months.

### Follow-up and Assessments

The patients were routinely followed up in the outpatient clinic. For patients who couldn't visit the clinic, they were advised to have the radiographs taken at the local hospital and mail them to our hospital. Implant loosening and component migration were radiographically evaluated; infection was assessed in light of serum erythrocyte sedimentation rate and C-reactive protein; and hip function was rated in terms of the Harris Hip Score (HHS) [14]. The horizontal and vertical migration of the COR were judged by using the method described by Peters et al. [15], and a high hip center was determined when it was located 35 mm above the interteardrop line [16]. Radiographic loosening was defined as cup migration of > 6 mm, abduction angle of > 10°, or progressive radiolucency in the 3 classic acetabular zones as described by DeLee and Charnley [17]. For the porous-coated acetabular component, the radiographic signs of bone ingrowth were evaluated according to the following criteria formulated by Moore et al. [18]: (1) the absence of radiolucent lines; (2) the presence of supero-lateral buttresses; (3) the presence of medial stress shielding; (4) the presence of radial trabeculae; and (5) the presence of infero-medial buttresses. When three or more signs were present, the positive predictive value of the radiographic test was 97 %; the sensitivity was 90 % and the specificity was 77 %. Postoperative complications were recorded on real time basis.

The primary endpoints included the clinical or radiographic evidence of acetabular loosening or both requiring a major revision surgery. The secondary clinical endpoints included the causes and complications that required a revision surgery. All chart reviews were performed by the same surgeon who was not involved in the treatments.

### Statistical Analysis

Statistical tests were carried out using SPSS 18.0 software package (SPSS Inc., Chicago, Illinois). The statistical significance was set at a *P* < 0.05. Categorical data, such as frequency and percentage, were analyzed using descriptive statistics. The continuous data were presented as the mean and range. Mann-Whitney U test was performed to examine any difference between the preoperative and postoperative HHS and the change of the COR. Using the revision surgery as an event, a survival analysis was used to report the survivorship of the multicup construct.

## Results

Fourteen patients (66.7 %) visited the office for complete clinical and radiographic assessment and 7 patients (33.3 %) only had a telephone evaluation for the HSS score and took radiographs in their local hospital. The mean follow-up period lasted 31 months (range, 18–57 months). We reconstructed 16 severe protrusio acetabular defects using 2 porous cups, and 5 defects employing 3 porous cups. We performed “cup-in-cup” technique in 7 patients, “cup-on-cup” one in 12, and “hybrid cup” technique in 2. The techniques were defined according to the type of composition of the shells. The patients' demographics and surgical characteristics are shown in Table 1. The operation time was defined as the time frame from the start of skin incision to the end of implantation (mean, 118 min; range, 85–235 min). The mean blood loss during revision surgery was 967 mL (range, 300 mL–3000 mL). The average intraoperative transfusion was 2.5 units (range, 0–8 units) of packed red blood cells and autotransfusion of 310 mL (range, 0 mL–1200 mL). The information of used implants in the current study is presented in Table 2.

**Table 2:** The information of used implants in current study

Patient No.	Paprosky Classification	No. of cups	Type of composition	Medial shell (mm)	Middle shell (mm)	Lateral shell (mm)	Additional Augmentation (mm)
1	IIC	2	cup-on-cup	52 (T)	None	58 (T)	None
2	IIIB	2	cup-in-cup	58 (T)	None	50 (T)	None
3	IIIB	2	cup-in-cup	68 (T)	None	64 (T)	58 × 15;48 × 10
4	IIIB	2	cup-on-cup	62 (T)	None	64 (T)	50 × 10;62 × 20
5	IIC	2	cup-on-cup	62 (T)	None	66 (T)	None
6	IIIB	2	cup-on-cup	62 (T)	None	66 (T)	None
7	IIC	2	cup-on-cup	54 (T)	None	64 (T)	None
8	IIIB	2	cup-on-cup	60 (T)	None	64 (T)	None
9	IIIB	2	cup-on-cup	64 (T)	None	66 (T)	None
62	IIC	2	cup-in-cup	62 (T)	None	58 (D)	60 × 15
11	IIIB	3	hybrid cup	66 (T)	54(T)	62 (T)	None
12	IIC	3	cup-in-cup	70 (T)	64(R)	58 (TRI)	None
13	IIIB	3	cup-in-cup	62 (T)	58(T)	54 (TRI)	None
14	IIIB	3	hybrid cup	60 (T)	56(R)	58 (T)	None
15	IIC	3	cup-in-cup	70(T)	60(R)	54 (R)	None
16	IIC	2	cup-on-cup	54 (T)	None	70 (T)	None
17	IIC	2	cup-on-cup	62 (T)	None	68 (T)	None
18	IIIB	2	cup-in-cup	70 (T)	None	62 (T)	60 × 15
19	IIIB	2	cup-on-cup	52 (T)	None	62 (T)	None
20	IIIB	2	cup-on-cup	58 (T)	None	66 (T)	None
21	IIC	2	cup-on-cup	54 (T)	None	62 (T)	None

*T* Zimmer, *TM* Tantalum revision shell, *R* mith&nephew, R3 Multi-hole hemispherical stiktite coated shell, *TRI* Zimmer, Trilogycontiuum acetabular system shell with cluster holes porous, *D* Depuy, Gription

### Clinical Outcomes and Complications

The HHS improved from 37.0 ± 7.1 (range, 24.3–47.7) preoperatively to 74.8 ± 8.5 (range, 57.2–87.6) after 1 year (*P* = 0.0001), and stayed at the level till the final follow-up (mean, 76.4 ± 9.0; range, 55.1–90.1).

The follow-up did not revealed any hip dislocation, infection, sciatic nerve injuries or periprosthetic fractures. Intramuscular calf vein thrombosis was reported in 2 (10 %) patients. Ankle dorsiflexion exercise was intensified after short-term administration of rivaroxaban. The vessels in thrombosis cases were in reperfusion 15 days and 21 days after surgery, respectively. In one patient, erythrocyte sedimentation rate and C-reactive protein level were found to be elevated 6 months after surgery. However, the patient had no symptoms associated with hip infection. The values were within the normal ranges after administration of levofloxacin for 2 weeks.

### Radiographic Results

The mean preoperative values of cranialization and lateralization of the COR were 35 mm (range, 26 mm–46 mm) and 11 mm (range, 15 mm–44 mm), respectively, while the mean postoperative values were 17 mm (range, 12 mm–23 mm) and 25 mm (range, 20 mm–31 mm), respectively (*P* = 0.0001). Preoperatively, a high hip center was observed in 12 patients. All migrations were corrected. At the latest follow-up, the radiographical examination showed that acetabular components were all stable without migration. Non-progressive acetabular radiolucencies were observed in no more than two zones in 2 patients. A total of 18 patients (86 %) satisfied at least 3 of the 5 criteria associated with bone ingrowth (as defined by Moore et al. [18]) and showed reliable osseointegration. No patient required acetabular component revision due to aseptic loosening of the multicup construct.

## Discussion

For the patients with the major protrusio acetabular defects (Paprosky IIC- Paprosky IIIB), the multicup reconstruction represents a simplified arthroplastic procedure that can be completed within a short period of time, with reduced blood loss. The multicup construction can effectively achieve a stable fixation and reduce the cranial migration of the hip COR, resulting in a good hip function.

With the hip that has severe protrusio acetabular defects, the conventional augmentation is often performed in a small operating space and depends on the contour of the defect. The drawbacks include prolonged operative time and increased blood loss, which are associated with an increased mortality rate [19]. Such uneven augmentation may lead to an increased micromotion and decreased bone ingrowth.

The double-cup reconstruction was first reported by Blumenfeld et al. [13] in 2012. The operative method involved two tantalum acetabular shells that were cemented with the “cup-in-cup” technique. They treated 7 patients and they had no loosening or migration after 2 years, and the horizontal and vertical offsets improved significantly. Webb et al. [20] treated 20 patients with acetabular loosening by using the “cup-on-cup” technique without acetabular revision. The hip COR was restored to an average of 23 mm above the interteardrop line. Loppini et al. [21] treated 9 cases of Paprosky type IIIB and 7 type IIIA acetabular defects by employing 2 TM cups. The HHS, leg length discrepancy, and COR position significantly improved after 2 years. Sculco et al. [22] reconstructed 57 severe acetabular defects using the cup-cage technique and reported a mean HHS of 72, with a dislocation rate of 7 % after 2 years. Gehrke et al. [23] reconstructed 46 acetabular defects using porous tantalum shells and augments and achieved a mean postoperative HSS of 82, with the dislocation rate being 5 % after 4 years.

Our technique has several advantages. First, directly pressing a hemispherical tantalum cup into the early cup simplified the surgical procedure. Second, the lateral cup was totally covered by the medial tantalum TM shell with an annular support, which allows the cup to be placed at the desired anteversion and abduction with a 10°- elevated-rim acetabular liners even in the most difficult cases. Third, the acetabular shells were implanted one by one based on the migration of the anatomical COR, which decreased the incidence of impingement, abductor tension, and lower limb discrepancy. Fourth, additional bone grafting, as needed, provides more osseointegration to achieve a long-term biological fixation of the prosthesis. Fifth, the biomechanical property of the porous tantalum acetabular components is associated with a much higher porosity, a modulus of elasticity similar to subchondral bone, and a very high coefficient of friction [24–27].

Two issues need to be addressed. First, the torque at the bone-medial shell interface is theoretically increased because the femoral head center is displaced inferiorly and laterally from the center of the medial shell, which may increase the risk of micromotion and affect bone ingrowth. Therefore, in the early postoperative period, full weight bearing should be avoided, but gradual and proper weight bearing could stimulate bone growth. Since osseointegration is the most important factor of this technique, we suggest that patients walk with toe-touch weight bearing within 6 weeks after revision and then with partial weight bearing for another 6 weeks. Loppini *et al*. [21] also advocated the same protocol. They reconstructed 16 Paprosky IIIA and IIIB acetabular defects using the “cup-on-cup” technique. The patients were allowed to walk with crutches and 30 % weight bearing on the first postoperative day. After 2 months, the patients were allowed to walk without crutches and with full weight bearing. The radiolucent line in zone 1 did not deteriorate in one hip after 6 years. The second issue is fixation strength of a porous shell cemented into the medial tantalum TM shell. A biomechanical study examined the pull-out and torsional fixation strength of cobalt chromium (CoCr) alloy acetabular components cemented into the titanium alloy acetabular components under different conditions [28]. The results revealed that the fixation strength of metal liners cemented into shells was comparable to that of the commonly-used locking mechanisms of polyethylene liners fixed into the metal acetabular shells, suggesting that the strength is sufficient for clinical application. Most of the shells used in our study were of tantalum TM structure, which, theoretically, might provide more fixation strength, given the characteristics of tantalum. In a series of 38 patients, Gabor et al. [29] performed the acetabular construction including a monoblock dual mobility cup cemented into a fully porous metal shell. No patients needed reoperation due to loosening of the acetabular component after 7 months, and the HHS improved from 50 ± 12 to 78 ± 11. Unlike cup-in-cup construct, the mechanism of cup-on-cup technique is press-fit fixation. Therefore, the fixation strength of this construction mainly relies on the lateral support rim.

What is more, the combination of multicups varied with patients, including the use of a tantalum TM shell (Zimmer Biomet, Warsaw, IN), an R3 multi-hole hemispherical StikTite-coated shell (Smith & Nephew, London, United Kingdom), Trilogy Continuum Acetabular System (Zimmer Biomet, Warsaw, IN) and the Gription TF shell (DePuy Synthes, New Jersey, NJ, United States), and the combination was related to the pattern of bone defects encountered and the patients’ financial status. Even so, it was noted that regardless of the combination of shells used, the tantalum TM shell served as a buttress cup in all cases due to its superior bone ingrowth characteristics. Moreover, it should be emphasized that if the “cup-on-cup” technique is selected according to the status of the acetabulum ring, then the tantalum TM shell should also be the first option. However, if the “cup-in-cup” technique is used, other shells with a porous design could be candidate implants due to their affordable price and rigid fixation by cement.

Our study has several limitations. First, the sample size was small since the cases were infrequent. Larger, multicenter studies are needed to further ascertain the results. Second, the follow-up period was short for this complex surgery. A longer follow-up period may improve the identification of a successful osseointegration. Third, this retrospective study may potentially cause uncontrolled selection biases. Last, there was no control group in this study since this issue is rare in clinical practice.

## Conclusions

The multicup reconstruction technique, a simplified version of reconstruction procedure, was used for managing the severe protrusio acetabular defects and could achieve a rigid fixation and high survival rate.

## Data Availability

This study was carried out in the First Affiliated Hospital of Xinjiang Medical University (137 South LiYuShan Road, Urumqi, Xinjiang, China). The datasets used and/or analyzed during the current study are available from the corresponding author on reasonable request..

## Abbreviations

TM: Trabecular metal
THA: Total hip arthroplasty
COR: Center of rotation
HSS: Harris hip score
